# Supplementary material for: Reduced Number and Immune Dysfunction of CD4+ T Cells in Obesity Accelerate Colorectal Cancer Progression
Source: Cells. 2022 Dec 25;12(1):86. doi: 10.3390/cells12010086 (PMC9818365; doi:10.3390/cells12010086)
Supplement: Supplementary file 1 [file cells-12-00086-s001.zip › cells-1865504-supplementary.pdf]

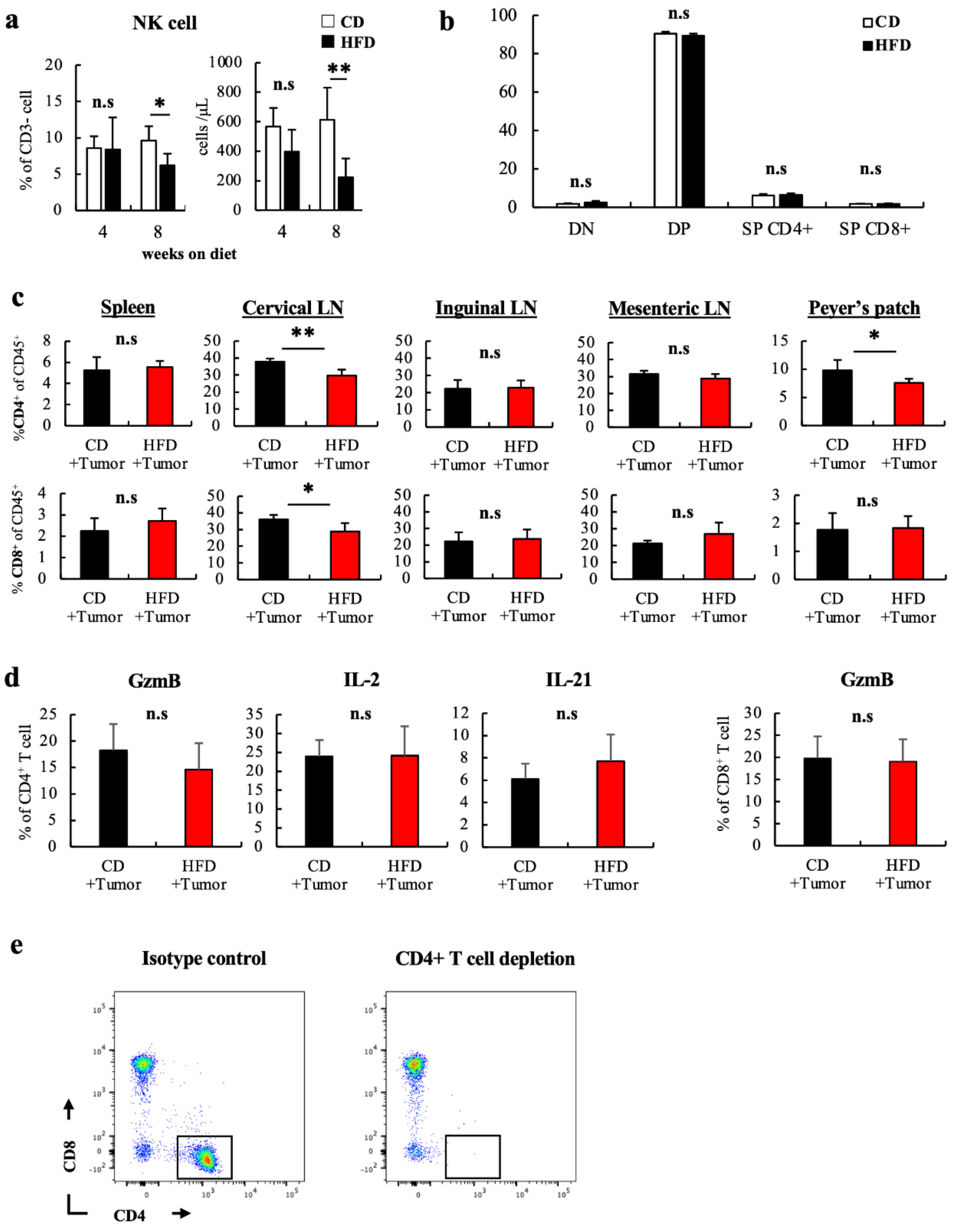

Figure S1. **(a)** Blood sample was obtained from the buccal vein, and changes over time in the number and percentage of NK cells in the peripheral blood were analyzed at 4 and 8 weeks. **(b)** Percentage of T cells in CD45+ cells in thymus at 12 weeks. **(c)** Percentage of T cells in CD45+ cells in secondary lymphoid tissues at

12 weeks of tumor-bearing mice. **(d)** The percentage of GzmB+, IL-2+, and IL-21+ of T cells in the tumor following stimulation with PMA+ ionomycin for 6 h is shown. **(e)** Depletion of CD4+ T cells in the blood was confirmed the day after antibody administration. The data are expressed as the mean ± standard deviation (S.D)。 * $P < 0.05$, **$P < 0.01$, ***$P < 0.005$.

Table S1. List of antibodies used.

Antibody	Source	Clone	Catalog Number	Dilution
FITC anti-mouse CD45.2 Antibody	BioLegend	104	Cat #109805	1/100
PE anti-mouse CD8a Antibody	BioLegend	S18018E	Cat #162303	1/100
PE anti-mouse CD107a Antibody	BioLegend	1D4B	Cat #121612	1/100
PE anti-mouse CD62L Antibody	BioLegend	MEL-14	Cat #104408	1/100
PE anti-mouse TNF-α Antibody	BioLegend	MP6-XT22	Cat #506306	1/100
PE anti-mouse GzmB Antibody	BioLegend	QA16A02	Cat #372208	1/100
PE/Cy7 anti-mouse CD3e Antibody	BioLegend	500A2	Cat #152314	1/100
PE/Cy7 anti-mouse KLRG-1 Antibody	BioLegend	2F1/KLRG1	Cat #138415	1/100
APC anti-mouse PD-1 Antibody	BioLegend	29F.1A12	Cat #135209	1/100
APC/Cy7 anti-mouse CD3e Antibody	BioLegend	500A2	Cat #152323	1/100
APC/Cy7 anti-mouse CD8a Antibody	BioLegend	53-6.7	Cat #100714	1/100
APC/Cy7 anti-mouse CD44 Antibody	BioLegend	IM7	Cat #103028	1/100
PerCP/Cy5.5 anti-mouse CD4 Antibody	BioLegend	GK1.5	Cat #100434	1/100
PerCP/Cy5.5 anti-mouse NK1.1 Antibody	BioLegend	S17016D	Cat #156526	1/100
PerCP/Cy5.5 anti-mouse IFN-γ Antibody	BioLegend	XMG1.2	Cat #505822	1/100
Pacific Blue anti-mouse CD4 Antibody	BioLegend	GK1.5	Cat #100427	1/100
Pacific Blue anti-mouse CD45.2 Antibody	BioLegend	104	Cat #109820	1/100
Brilliant Violet anti-mouse IL-2 Antibody	BioLegend	JES6-5H4	Cat #503825	1/100
